# Design of the Artificial Intelligence Vocal System for Music Education by Using Speech Recognition Simulation

**DOI:** 10.1155/2022/5066004

**Published:** 2022-09-27

**Authors:** Junqing Bai

**Affiliations:** Shanxi Vocational University of Engineering Science and Technology, Jinzhong, Shanxi 030619, China

## Abstract

At present, computer technology is not limited to the network but to many aspects of spread out, including artificial intelligence technology, signal processing technology, speech recognition technology, and so on which have been better developed, and music teaching has also obtained certain achievements. If the detected speech data have no other clutter in the speech, then the endpoint detection can achieve ideal results. The music education system includes the development of four dimensions: WEB terminal, mobile terminal, web interface, and database. Using the powerful functions of computers, such as computing functions and processing functions, combined with advanced intelligent technology and related equipment, the intelligent learning model based on computer technology is established. It can be applied in college teaching, combined with the actual needs of current college music teaching, corresponding to the curriculum and teaching plan issued by the school, combined with the design of online learning system and reasonable use of computer technology to build a vocal music education system. The intelligent system can improve the requirements of vocal music education management level, teaching organization ability, and teaching platform operation ability.

## 1. Introduction

The realization of everything is a process of unifying the theoretical foundation and practical activities, as is the music education system. At present, since the ITS system does not have its complete theoretical foundation, it will bring a series of development problems to the vocal music system [1]. In this article, we mainly discuss the study of music education system from three major aspects: the development process of music education, the comparison of cross-regional development, the goals, and tasks of music education, and the vocal music system developed according to the characteristics of music education [2]. Among them, for the music education system, in the research of this article, we mainly use J2EE technology, UML modeling, JAVA language, and web programming technology based on the current music teaching needs, combined with music theory preparation to carry out relevant practices of music education activities [3]. The music education system with J2EE technology as the core includes the design and configuration of system management skills, the design and configuration of music resource management skills, and the design and configuration of music course management skills [4]. The realization of speech recognition function is the result of the unification of acoustics, computer science, artificial intelligence, and other technologies [5]. At present, speech recognition technology still has a lot of room for improvement. For example, in real life, when we communicate with others, because of the flexibility of language and the language rules established by the society, we can flexibly pause and delete according to our own expression needs, but when we communicate with machines, if it is for a single word or more words, then we must pause when necessary; otherwise, the machine will fail to recognize errors [6]. At present, the development of the Linux operating system is unstoppable, and the development of graphical user interfaces under its system has become a hot topic at the moment [7]. The graphical user interface is based on the processing of various icons, using the powerful functions of the computer, such as calculation functions and processing functions, , combined with advanced intelligent technology, related equipment, etc., to put the operator and the machine in the same high level and to achieve the purpose of coordination and cooperation between the two so as to bring users a simple and clear experience in the operation process [8]. The graphical user interface requires it to have the basic functions of small footprint, high performance, and high security. It can be seen that the graphical user interface has a high position in the software system. Of course, due to the lack of its own hardware, its changes cannot be controlled.

## 2. Related Work

Literature shows that the design principle of modern embedded system is a series of work carried out on the basis of mutual cooperation between software and hardware [9]. For example, the powerful data processing capability of embedded system is embedded in the microprocessor and digital signal, realized on the basis of mutual cooperation of processors. The design of the traditional embedded system really separates the two. First, the two parts of software and hardware are correspondingly processed, and then the hardware part is processed first, and the software performs corresponding operations afterwards. To some extent, compared with modern embedded systems, the operation process of traditional embedded systems is extremely complex and time-consuming. Literature shows that XML—a metadata language can be described in a unified and convenient way, and the result can be structured data, and the system can perform related operations on data across multiple platforms. The final result is more meaningful [10]. XML is not just a service for certain programs, and it has all versatility. The program is easy to execute and can be changed according to user needs. It is proposed in the literature that communication between people and computers can be realized by simulating the dialogue between people [11]. This is a case of applying speech recognition technology to the field of communication and bionics for the benefit of society. For example, for people with physical disabilities, when they are inconvenient to operate with their hands, the emergence of voice recognition can completely get rid of the operation of computer hardware. When this operation is applied to the factory floor, it can be operated manually [12]. Significantly reducing labor costs will help companies realize industrial transfer; in addition, language recognition technology can convert speech into words and words into sounds, which greatly facilitates our lives. According to the literature, from the most fundamental structure, artificial neural network is a learning and training method that simulates neural complexity [13], and the exploration of artificial neural network is completed through continuous deep learning to deal with the complex relationship of the data, so as to carry out mathematical modeling algorithms. Literature shows that the research on neural networks dates back to 2006 [14]. At that time, some people tried to explore with traditional training algorithms, but the results were not successful. The literature shows that most of the students majoring in music in colleges and universities in our country are students whose main major is vocal music learning. To sum up, vocal music education in colleges and universities needs a high level of teaching [15]. However, from the current teaching situation, we can see that music education in colleges and universities still has a lot of negligence: backward teaching materials, aging teaching equipment, one-sided teaching management, unscientific teaching arrangements, and teaching methods that are difficult to stimulate students' interest in learning. These are the legacy of the old teaching. We are also facing another problem of lack of teachers. In the field of vocal music education and teaching, we urgently need to improve. The literature shows that the subject of music is different from the education and teaching of other cultural courses [16]. It has its own unique needs for teachers' teaching level and teaching equipment. In accordance with the current development needs, many schools have optimized vocal music education, adjusting teaching plans, teaching equipment, teaching programs, and other aspects.

## 3. Research on Language System

### 3.1. Basics of Speech Recognition

**Figure 1:**
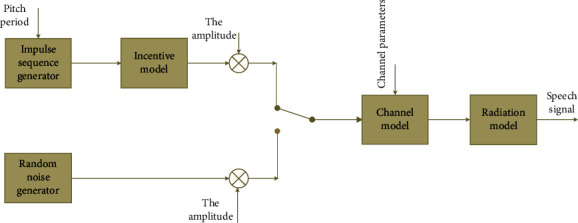
The relationship between the speech signal models.

Next, based on the embedded language system, a comprehensive analysis of the linear model of its speech signal and important technologies in language recognition technology will be carried out. Voice signal is defined from the human physiological level, and when a person is making a sound, the airflow causes an impact on the vocal tract, making the person's vocal cords to vibrate, and then the airflow can flow out of the oral cavity or nasal cavity according to the characteristics of the pronunciation. The model is built based on the appearance of the voice signal. Because of the one-way nature of the path, the model also presents this linear pattern. The path is generated for it, as shown in Figure 1:

We can introduce them one by one as follows:

(1) The study of the excitation model points out that when people make different sounds, the excitation generated by the speech signal is also different. When *g*_1_ and *g*_2_ are both close to 1, then
  (1) $$\begin{array}{c} E\left(z\right)=\frac{A_{v}}{1-z^{-1}}\text{.} \end{array}$$
*A*_*v*_ represents energy which is represented as
  (2) $$\begin{array}{c} U\left(z\right)=G\left(z\right)E\left(z\right)=\frac{A_{v}}{1-z^{-1}}\times \frac{1}{\left(1-g_{1}z^{-1}\right)\left(1-g_{2}z^{-1}\right)}\text{.} \end{array}$$
(2) When the pronunciation is different, the vocal tract changes differently. There are two main changes: when the vowel sounds, the oral environment is relatively stable. When consonants are pronounced, high-pressure waves appear in the vocal tract. We usually use formant models to indicate these changes. The resonance peak can be represented by
  (3) $$\begin{array}{c} V\left(z\right)=\frac{1}{\sum_{i=0}^{p}a_{i}z^{-i}}\text{.} \end{array}$$
(3) The voice signal is essentially a sound pressure wave. In the process of voice signal generation, it will have a specific speed through the vocal cords. These two inverse factors are called radiation impedance. It represents the interaction between the lips. Formula (4) is the relationship between the lip area and the head when the speech signal is generated:

(4) $$\begin{array}{c} z_{L}\left(\Omega \right)=\frac{j\Omega L_{r}R_{r}}{R_{r}+j\Omega L_{r}}\text{.} \end{array}$$

The radiation model is given as

(5) $$\begin{array}{c} R\left(z\right)=1-\left(rz^{-1}\right)\text{.} \end{array}$$

Among them, *r* ∼ 1.

In summary, the linear model of the speech signal can be inspired by the three examples cited above:

(6) $$\begin{array}{c} H\left(z\right)=U\left(z\right)V\left(z\right)R\text{.} \end{array}$$

**Figure 2:**
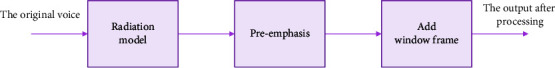
Pretreatment process.

Because most of the initial speech is an original signal, we cannot directly get the information we need, so it needs to be preprocessed.  Figure 2 shows the preprocessing steps.

Next, we explain the pretreatment process in detail.

**Figure 3:**
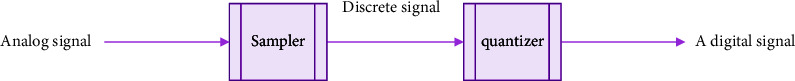
Analog-to-digital conversion process.

When we store sound information in the computer, we need to perform voice signal ⟶ digital signal, as shown in Figure 3:

Analog audio⟶digitalization can be shown as

(7) $$\begin{array}{c} x\left(n\right)=x_{n}\left(n-T\right)\text{.} \end{array}$$

In this formula, *T* is voice sampling period; electrical signal ⟶ binary code.

The power spectrum of the speech signal is not completely invariant, and it has an inverse correlation with energy. The higher the energy, the smaller the value. Therefore, most of the energy is concentrated in the low frequency part of the signal, but this phenomenon can easily cause the high-frequency part of the signal to be unrecognizable, so our solution is to use pre-emphasis to process the sampled and quantized voice signal. This can raise the high-frequency part of the signal and break the energy tilt phenomenon. The same conditions can be used to solve the frequency of the signal:

(8) $$\begin{array}{c} H\left(z\right)=1-uz^{-1}\text{.} \end{array}$$

The voice signal, taking humans as an example, is a signal that changes all the time and does not have stability, but its changes are small in a very short period of time. So we can regard it as a steady-state signal in some processing. But in the case of extremely short signal, if there is a transition between two adjacent syllables or between the first letter and the last, the sound will change between two adjacent tracks. In this case, its characteristic parameters may vary greatly. Like the pre-emphasis above, we need to place the voice signal in the same part for processing, and the method used is frame processing. In the framing operation, *x*(*n*) × *w*(*n*), and then the derived results are weighted. The following formula (9) is the windowed voice signal which is given as

(9) $$\begin{array}{c} y\left(n\right)=\overset{+\infty }{\underset{-\infty }{\sum }}x\left(m\right)w\left(-n\right)\text{.} \end{array}$$

Speech analysis usually uses three types of window function, such as Hamming window, Hanning window and rectangular window

**Figure 4:**
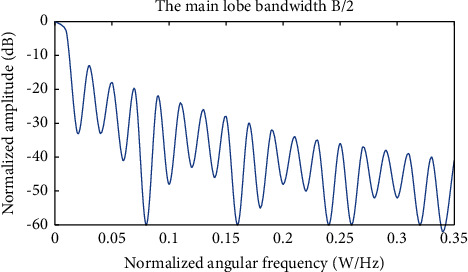
Amplitude frequency response of the window function.

Figure 4 shows the amplitude frequency response of the window function, *a* represents the attenuation of the first lobe, and *B* represents the width of the main lobe. Then compare the characteristics of the window function.  Table 1 shows the characteristic parameters:

### 3.2. Endpoint Detection

It can be seen from the table that closing A at the first side line of the window function is the largest. This shows that the leakage of the spectrum is much smaller than the other two, and the characteristics of passing through the blocking will be better. The system uses the clear function to identify the smoothing requirements of the signal. The frame length reported here is 256, and the frame shift is 80.

In a complete language, the starting point and focus of the language also need to be tested, which uses a program called endpoint detection.

(1) Combined with the above, in the voice data, the *i*-th frame data is *y*_*i*_(*n*), and *y*_*i*_(*n*) needs to meet:
  (10) $$\begin{array}{c} y_{i}\left(n\right)=w\left(n\right)*x\left(\left(i-1\right)*frameinc+n\right),1\leq n\leq L,1\leq i\geq f_{n}\text{.} \end{array}$$
The short-term energy of the *i*-th frame is
  (11) $$\begin{array}{c} E_{i}=\overset{L-1}{\underset{n=0}{\sum }}y_{i}^{2}\left(n\right),1\leq i\leq n\text{.} \end{array}$$
The following formula (12) is the average amplitude of speech:
  (12) $$\begin{array}{c} M\left(i\right)=\overset{L-1}{\underset{n=0}{\sum }}y_{i}\left(n\right)\text{.} \end{array}$$
(2) After the speech recognition technology is recognized, data ⟶ digital signal. It can be seen that after recognition, the speech signal has a sign change. The total number of these sign changes is called the short-term average zero-crossing rate. It is calculated as

(13) $$\begin{array}{c} zcr\left(i\right)=\frac{1}{2}\overset{L-1}{\underset{n=0}{\sum }}\left|\text{sgn}\left[y_{i}\left(n\right)-\text{sgn}\left[y_{i}\left(n-1\right)\right]\right]\right|,1\leq i\leq f_{n}\text{.} \end{array}$$

In the following formula sgn is a symbolic function:

(14) $$\begin{array}{c} \text{sgn}\left[x\right]=\left\{\begin{array}{cc} 1\text{,} & x\geq 0\text{,}\\ -1\text{,} & -1,x<0\text{.} \end{array}\right. \end{array}$$

**Figure 5:**
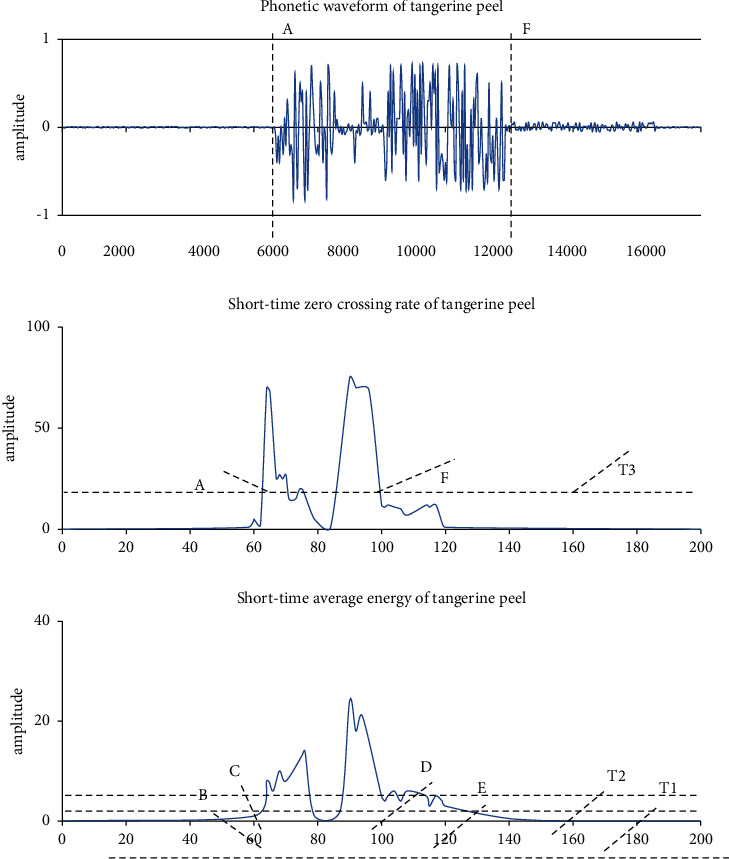
Schematic diagram of the two-level judgment method.

In the process of detecting voice endpoints, it is likely that the detection is not accurate enough. Therefore, we will introduce a new language endpoint detection method, that is, the first proposed two-layer critical price method. This method uses short-term average English based on exchange rate and short-term energy, and the principle is that Chinese finals contain vowels and have high energy, which will have higher frequencies depending on the pronunciation and will have a higher short-term repetition rate according to different letters, which can be distinguished according to two characteristics such as the uppercase letters of the sound and the last Chinese syllable. The two-layer critical value method is shown in Figure 5, which is embodied on the basis of the second judgment method.

**Figure 6:**
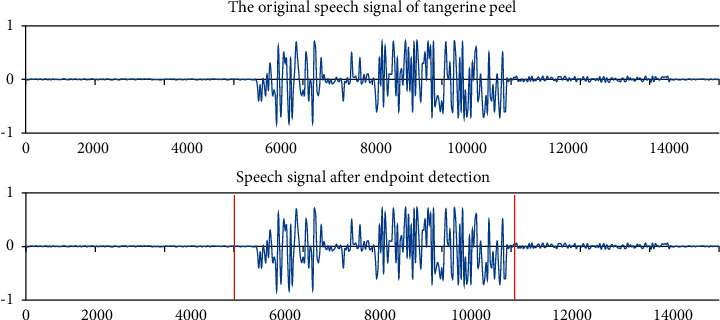
“Tangerine peel” endpoint detection results.

In Figure 6, the start and end points of the speech are marked as A and F, respectively. The endpoint detection procedure are as follows: (1) the pre- and window setting of the voice signal; (2) the voice after the stage will calculate the ratio of the short-term average energy and the short-term average zero transition; (3) finally, the critical price comparison and endpoints analysis; and (4) perform the original terminal monitor for the sound signal. The endpoint detection results are shown in Figure 6.

Noise will have a certain degree of influence on speech detection. If the detected voice data have no other messy sounds in the voice, then the endpoint detection can achieve the desired result. If the speech is superimposed with some messy sounds, that is, noise, then its recognition will be hindered to a certain extent.

Comparing the figure above, it can be clearly seen that there is a big difference in the short-term average zero-crossing rate between the two. This also shows that the voice signal under the influence of noise is affected as a result and even caused an error in the detection structure:

(15) $$\begin{array}{c} y\left(n\right)=\text{med}\left(x\left(n-L\right),x\left(n-L+1\right),\ldots ,x\left(n\right),\ldots ,x\left(n+L\right)\right)\text{.} \end{array}$$

Formula (15) can self-clear some speech endpoints that have no effect, leaving only the effective endpoints, and after they are cleared, the existence value of the sound paragraphs without energy fluctuations will not have a major impact. Of course, in actual applications, we will make changes in a certain link according to actual needs. For example, in some special cases, we need to change the maximum value of dual-threshold endpoint detection to make it meet our needs.

The energy min is

(16) $$\begin{array}{c} amp0=1.2*\text{mean}\left(\text{amp}1\right)\text{.} \end{array}$$

Energy max = min × 1.5, and the threshold of zero-crossing rate is

(17) $$\begin{array}{c} zcr1=0.8*\text{mean}\left(\text{zcr}1\right)\text{.} \end{array}$$

Because our research was conducted in a laboratory environment, it is inevitable that our voice collection will have noise superimposition, which will cause the relative value of noise to be higher. In order to solve this problem, in the collected voice samples, Gaussian white noise is added, and the next experiment will continue to use the original speech as the research object.

Because of the complexity of the speech signal, we are prone to ambiguities in the research process. At this time, we often use a large number of parameters to explain, but when the parameters reach a certain number, we may violate the essence of the research. On the contrary, it will cause another complicated situation. Therefore, using the least parameters to describe the most comprehensive experimental results is an important aspect of our speech signal research. When solving this problem, we have used the time domain, and its description of the experimental results is pass, but it still does not get rid of the phenomenon of disordered parameters. So based on this phenomenon, we later discovered a new method; that is, in speech recognition, different speech generation models are described separately to avoid the mixing of the two, that is, to separate the speech signal. The method is to conduct research on the basis of the nature of the speech signal, which effectively circumvents many complicated analyses and makes the feature parameters come out.

Linear predictive analysis is mainly used in voice recognition, speech synthesis, and speech analysis technology used in coding. The basic principle of linear predictive analysis is that because the collection of various speech signals is in the same space, they will be similar to a certain extent. Therefore, in order to avoid the interference of results caused by this phenomenon, we can pay attention to some samples before sampling, or we can repeat the test and analyze the results of multiple tests. It is also possible to use the LPCC analysis method for processing. The calculation step of this method is

(18) $$\begin{array}{c} H\left(z\right)=\frac{1}{A\left(z\right)}=\frac{1}{1-\sum_{j=1}^{p}a_{j}z^{-j}}\text{.} \end{array}$$

Cepstrum *h*′(*n*) can be given as

(19) $$\begin{array}{c} \ln\;H\left(z\right)=\overset{\infty }{\underset{n=1}{\sum }}h\prime \left(n\right)z^{-n}\text{.} \end{array}$$

Formula (18) when combined with formula (19), we can get

(20) $$\begin{array}{c} \frac{\partial }{\partial z^{-1}}\ln\frac{1}{1-\overset{p}{\underset{k=1}{\sum }}a_{k}z^{-k}}=\frac{\partial }{\partial z^{-1}}\overset{\infty }{\underset{n=1}{\sum }}h\prime \left(n\right)z^{-n}\text{,} \end{array}$$

which is,

(21) $$\begin{array}{c} \overset{\infty }{\underset{n=1}{\sum }}h\prime \left(n\right)z^{-n}=\frac{\overset{p}{\underset{k=1}{\sum }}a_{k}z^{-k+1}}{1-\overset{p}{\underset{k=1}{\sum }}a_{k}z^{-k}}\text{.} \end{array}$$

So we have

(22) $$\begin{array}{c} 1-\overset{p}{\underset{k=1}{\sum }}a_{k}z^{-k}\overset{\infty }{\underset{n=1}{\sum }}nh\prime \left(n\right)z^{-n+1}=\overset{p}{\underset{k=1}{\sum }}ka_{k}z^{-k+1}\text{.} \end{array}$$

According to formula (22), the relationship between *h*′(*n*) and *a*_*k*_ can be obtained as

(23) $$\begin{array}{c} \left\{\begin{array}{c} h\prime \left(0\right)=0\text{,}\\ h\prime \left(1\right)=a_{1}\text{,}\\ h\prime \left(n\right)=a_{n}+\overset{n-1}{\underset{k=1}{\sum }}\left(1-\frac{k}{n}\right)a_{k}h\prime \left(n-k\right),1\leq n\leq p\text{,}\\ h\prime \left(n\right)=\overset{p}{\underset{n=1}{\sum }}\left(1-\frac{k}{n}\right)a_{k}h\prime \left(n-k\right),n>p\text{.} \end{array}\right. \end{array}$$

In view of the complex parameter problems in the above table, we then introduce the Mel cepstrum coefficient MFCC, which is our most commonly used parameter in the current speech recognition system. It is mainly based on the model development process that people obtain speech signals in the process of hearing and has strong characteristics of identifying noise and speech.

### 3.3. Research on Embedded Systems

XML is a conversion medium for data conversion of language signals. It can realize the mutual conversion of language signals and language data under certain standards. Of course, it can also be used to leap across different platforms instead of constrained by one platform. XML is a universal standard. It is the same as the above. It is not only applicable to one mode but can also achieve leapfrogging under different platforms, avoiding the complexity caused by situational constraints.

It can be said that this module is another important part of the system, but it is no longer a purely technical limitation, and it includes a part of the embodiment of personal will. In this module, it mainly refers to the assembly of the user interface and the customization of its style. Therefore, affected by the designer and related research and development institutions, it will have some corporate colors and personal will. Its form is flexible, and it is no longer purely due to technical factors which are limited. Combined with the user interface customization module introduced above, for the assembly of the user interface, we only need to follow the respective group interface, and then connect with the corresponding user interface system.

### 3.4. Test for the Speech Recognition System

The software environment of the experiment is the Microsoft Visual C++ development environment under the windows operating system. This speech recognition system uses 10 Chinese words as commands.  Table 2 shows the no feedback speech recognition system recognition, and Table 3 shows the unmonitored VGC method recognition.

It can be seen from the above table that it has a good ability to respond when facing changes in the voice signal and can be adjusted appropriately according to the changes in the speaker, the environment, and certain objective conditions. So we can say that this detection method can be quantitatively copied in its own program, and the effect of batch learning is amazing.  Table 4 shows the monitoring of the results of VGC method recognition, and Table 5 shows the unmonitored TWA method recognition results.

## 4. Design and Research on the Artificial Intelligence Vocal Education System

### 4.1. Artificial Intelligence

From the above table, we can clearly see that the work learning efficiency of artificial intelligence is several to several tens of times than that of human work and learning. In the field where artificial systems have been used on a large scale, artificial intelligence systems already have the corresponding operating principles and have data processing imported into their instruction programs, so they only need to be able to work with power guarantees, and as long as there is no electricity limitation, it can continue to go on. At the same time, it has an obvious advantage. It does not have human consciousness, the quality of the working environment, the level of salary, and the good relationship between staff members. Bad things will not cause them to oppose and protest, and for certain areas that humans cannot reach, they can also replace humans in work. At the same time, to a large extent, there will be no uneven work results. Therefore, the emergence of artificial intelligence has begun to gradually replace people's positions in certain fields, which will bring greater changes to today's job changes. Of course, on the positive side, using artificial intelligence systems to replace humans will greatly reduce corporate expenditures, improve corporate economic efficiency, promote industrial transfer, and promote economic development.

The three parts of the model, view, and controller can be integrated and named as the MVC mode. This mode is usually used for software design, system development, and module repair, and it has a variety of styles in it. By design, the operator can use this ready-made service to carry out related operations, which greatly reduces the manual operation time. At present, the music education system we mainly use is WEB programming technology, which is the current mainstream development technology. It includes the development of four dimensions: WEB, mobile, web interface, and database.

IThis study is mainly based on J2EE technology and uses SQLServer2012 as the database development tool in the development of the database. At present, based on the actual situation, we mainly have two common online communication modes as follows:

1. *An online communication method similar to QQ and WeChat*. When we need to talk to someone, we can find the other person's dialog box on the relevant interface, and then have a one-to-one private chat. Of course, we can also open multiple dialog boxes at the same time and then communicate with multiple people in real time. When the user is temporarily offline, the pop-up interface will show the other party's situation. Once the other party is online, the dialog box in the information list will automatically sound a reminder.
2. *Another online communication is very similar to the forum mode*. We can open the corresponding interface to see others' answers under different types of questions. At the same time, we can also express our own opinions and put forward the problems that we urgently need to solve. We can also have online discussions with many users in the forum, and everyone can express their views, and finally everyone unanimously chooses the answer that best fits the topic.

### 4.2. Status Quo and Development of Vocal Music Teaching

The current university vocal music education curriculum system has many problems that are not adapted to the development of the times. Teaching materials are outdated, teaching equipment is aging, teaching management is one-sided, teaching arrangements are not scientific, and teaching methods are difficult to stimulate students' interest in learning. These are the legacy of old-fashioned teaching. At the moment, we are also facing another problem of lack of teachers.

The suggestion on how Chinese universities should improve the quality of vocal music classes is reform the traditional one-to-one courses and introduce small class teaching and group teaching methods, then students can help each other in the classroom, learn from each other, encourage each other, and influence each other. When encountering problems, analyze, review, and solve problems together, improve students' ability to analyze and solve problems, and cultivate students' ability to create music. Students are required to boldly express their opinions on the choice of vocal music works. In order to protect students' personalities and form a good teacher-student relationship, a relaxed educational environment should be established to stimulate students' love for music and art, and for students' future teaching work to provide models. In the vocal music classes of various schools, we must be brave to innovate, train students with solid professional qualities, and boldly reform the vocal music teaching model. Through teaching, students are exposed to a large number of works, and let them form their own vocal works of different styles. Experience the emotional elements and aesthetics contained in various vocal music works and explore the aesthetic value of vocal music. Students must not only be limited to simple learning in the learning process but also master the basic singing skills and also broaden students' horizons and enhance their perception and expression ability by focusing on acquiring and expressing the content of the song.

### 4.3. Design and Research of the Vocal Music Education System

Java not only provides a framework but also roughly agrees on the overall architecture of web applications. Moreover, a series of specifications have been made for this requirement, which is J2EE. The specification only provides the overall structure, process, and API, and the user implements these specifications. For example, Tomcat implements Web Container and its API. So to sum up, J2EE is a series of specifications related to web development on the Java platform. Companies such as Sun/Oracle/IBM/Red Hat provide the implementation of J2EE specifications. We generally use one or the other when we use Java for web development and multiple implementations. Correspondingly, our own code also follows the J2EE specification.

UML is an acronym for Unified Modeling Language called Standard Modeling Language. UML is a visual modeling language for software-intensive systems, often expressed in graphical form. Specifically, a typical UML diagram includes several blocks or block diagrams, connecting lines, and text representing additional information of the model of these elements. The main method of UML is not software design but software requirements analysis. UML can help analyze and execute software requirements and software design work. When using UML to analyze the software requirements, the learning threshold will be greatly reduced, and the grammatical complexity will also be reduced.

## 5. Conclusion

Speech recognition has been actively developed as a new method of human-computer interaction. This article introduces the operation process of each system function. The main functions of the system include system management function, music education management function, music course management function, and online communication function. The courses of ordinary colleges and universities are not rich, and the division is not detailed, and the curriculum setting is relatively scattered, unsupervised, and lacks a complete academic system, and there is no overall plan, so the range of choices for students is small. The existing music teaching programs in colleges and universities can no longer meet the new curriculum requirements, and there are still many problems, and it is difficult for students to learn to meet the requirements of the development of the times. For nonmusic major students, their music teaching is more of a formality that students cannot really learn more music knowledge. The teaching content combines general knowledge of music and singing ability. But the songs are arbitrarily selected, and it also contains songs by many singers and excellent folk songs. However, from the perspective of humanities and culture, there are few vocal works with broad meaning, ideology, artistry, and greater training value. Therefore, this aspect needs to be further optimized. At the same time, university vocal music teachers should also respond to this. Continuous optimization is needed, so we have to pay more efforts for the dissemination of music art.

## Figures and Tables

**Table 1:** First side lobe attenuation A and main lobe width B.

	Rectangular window	Hanning window	Hamming window
B	0.89 Δw	1.44Δw	1.30Δw
A/dB	13	32	43

**Table 2:** No feedback speech recognition system recognition results.

Tesp speech	(Each one has 50 test samples)									
	1	2	3	4	5	6	7	8	9	10
No.1	47	0	0	0	0	0	0	0	0	0
No.2	0	48	0	0	0	0	0	0	0	0
No.3	0	0	34	0	0	0	0	0	0	0
No.4	0	0	0	30	0	0	0	0	0	0
No.5	0	0	0	0	44	0	0	0	0	0
No.6	0	0	0	0	0	38	0	0	0	0
No.7	0	0	0	0	0	0	48	0	0	0
No.8	0	0	0	0	0	0	0	45	0	0
No.9	0	0	0	0	0	0	0	0	47	0
No.10	0	0	0	0	0	0	0	0	0	46

**Table 3:** Unmonitored VGC method recognition results.

Tesp speech	(Each one has 50 test samples)									
	1	2	3	4	5	6	7	8	9	10
No.1	50	0	0	0	0	0	0	0	0	0
No.2	0	48	0	0	0	0	0	0	0	0
No.3	0	0	45	0	0	0	0	0	0	0
No.4	0	0	0	43	0	0	0	0	0	0
No.5	0	0	0	0	42	0	0	0	0	0
No.6	0	0	0	0	0	47	0	0	0	0
No.7	0	0	0	0	0	0	47	0	0	0
No.8	0	0	0	0	0	0	0	48	0	0
No.9	0	0	0	0	0	0	0	0	48	0
No.10	0	0	0	0	0	0	0	0	0	48
	N	N	N	N	N	N	N	N	N	N

**Table 4:** Monitoring the results of VGC method recognition.

Tesp speech	(Each one has 50 test samples)									
	1	2	3	4	5	6	7	8	9	10
No.1	50	0	0	0	0	0	0	0	0	0
No.2	0	48	0	0	0	0	0	0	0	0
No.3	0	0	49	0	0	0	0	0	0	0
No.4	0	0	0	49	0	0	0	0	0	0
No.5	0	0	0	0	45	0	0	0	0	0
No.6	0	0	0	0	0	47	0	0	0	0
No.7	0	0	0	0	0	0	48	0	0	0
No.8	0	0	0	0	0	0	0	49	0	0
No.9	0	0	0	0	0	0	0	0	48	0
No.10	0	0	0	0	0	0	0	0	0	48
	N	N	N	N	N	N	N	N	N	N

**Table 5:** Unmonitored TWA method recognition results.

Tesp speech	(Each one has 50 test samples)									
	1	2	3	4	5	6	7	8	9	10
No.1	47	0	4	31	0	0	0	0	0	0
No.2	0	48	0	0	0	0	3	0	0	0
No.3	0	0	1	0	31	0	0	0	0	0
No.4	0	0	20	43	0	0	0	0	0	0
No.5	0	0	1	0	47	0	0	3	0	0
No.6	0	0	0	0	0	8	3	40	0	0
No.7	0	0	0	10	0	1	40	0	0	0
No.8	0	0	0	0	0	0	0	48	0	51
No.9	2	0	0	0	0	0	0	0	49	0
No.10	0	0	38	0	0	0	13	0	0	0
	N	N	N	N	N	N	N	N	N	N

## Data Availability

The data used to support the findings of this study are available from the corresponding author upon request.
